# Endocrine and Bone Complications in *β*-Thalassemia Intermedia: Current Understanding and Treatment

**DOI:** 10.1155/2015/813098

**Published:** 2015-03-05

**Authors:** Adlette Inati, MohammadHassan A. Noureldine, Anthony Mansour, Hussein A. Abbas

**Affiliations:** ^1^Department of Pediatrics, Rafic Hariri University Hospital, Beirut, Lebanon; ^2^School of Medicine, Lebanese American University, Byblos, Lebanon; ^3^Lebanese American University and University Medical Center-Rizk Hospital, Beirut, Lebanon; ^4^Department of Molecular and Cellular Oncology, University of Texas M. D. Anderson Cancer Center, Houston, TX 77030, USA

## Abstract

Thalassemia intermedia (TI), also known as nontransfusion dependent thalassemia (NTDT), is a type of thalassemia where affected patients do not require lifelong regular transfusions for survival but may require occasional or even frequent transfusions in certain clinical settings and for defined periods of time. NTDT encompasses three distinct clinical forms: *β*-thalassemia intermedia (*β*-TI), Hb E/*β*-thalassemia, and *α*-thalassemia intermedia (Hb H disease). Over the past decade, our understanding of the molecular features, pathophysiology, and complications of NTDT particularly *β*-TI has increased tremendously but data on optimal treatment of disease and its various complications are still lacking. In this paper, we shall review a group of commonly encountered complications in *β*-TI, mainly endocrine and bone complications.

## 1. Introduction

*β*-thalassemia, one of the most common monogenetic diseases worldwide, constitutes a group of hereditary blood disorders resulting from a defect in the synthesis of the *β*-globin chain [1, 2]. This defect causes a disproportionate ratio of alpha- and beta-globin chain synthesis leading to ineffective erythropoiesis (IE) and a chronic hemolytic anemia. Based on genetic and clinical features, *β*-thalassemia is divided into 3 distinct categories: thalassemia major, thalassemia intermedia, and thalassemia minor. Patients with *β*-thalassemia major (*β*-TM) harbor two defective copies of the *β-globin* chain and present during the first 2 years of life with a severe lifelong transfusion dependent microcytic anemia. People with thalassemia minor or the carrier state have one defective copy of the *β*-globin chain (heterozygous) and are usually clinically silent [1–3].

Beta thalassemia intermedia (*β*-TI) is a disease of intermediate severity where affected patients usually present with a later onset of microcytic anemia and milder clinical symptoms compared to *β*-TM. It belongs to the nontransfusion dependent thalassemia (NTDT) group which also includes *α*-thalassemia intermedia (hemoglobin H disease) and hemoglobin E/*β*-thalassemia (mild and moderate forms). It arises from a homozygous or a compound heterozygous mutation leading to partial suppression of beta-globin protein production. Three mechanisms are responsible for the milder phenotype of *β*-TI: inheritance of a mild or silent beta-chain mutation, coinheritance of *α*-thalassemia, and hereditary persistence of HbF, *δβ*-thalassemia, and G*γ* XMN1 polymorphism [2–4].

The clinical manifestations and complications of *β*-TI are unique and different from well treated *β*-TM patients but are similar to *β*-TM patients who are poorly transfused. Hence, it is plausible that the manifestation of *β*-TI are due to dyserythropoiesis. The disease is characterized by marked phenotypic heterogeneity with some patients remaining asymptomatic and maintaining a baseline hemoglobin range of 7–10 g/dL, while some others requiring transfusions due mostly to suboptimal growth and development, skeletal deformity, exercise intolerance, and declining hemoglobin levels because of progressive splenomegaly. Typical physical exam findings include growth retardation, thalassemic bone deformities, splenomegaly, and moderate to severe hepatomegaly [2, 3, 5].

The triad of chronic anemia, ineffective erythropoiesis, and iron overload characterizes *β*-TI and is mostly responsible for its clinical sequelae. Other disease complications include endocrinopathies, bone disorders, and end organ damage. Some complications as extramedullary hematopoiesis, leg ulcers, gallstones, thrombosis, and pulmonary hypertension, are rarely encountered in *β*-TM, but frequently seen in *β*-TI [2–4, 6, 7]. Older age and splenectomy have been shown to be independently associated with an increased risk of most *β*-TI disease-related complications [8, 9]. Much of the disease associated morbidity and mortality can be reduced with regular surveillance, early treatment, and follow-up in a comprehensive multidisciplinary setting.

Treatment of *β*-TI needs to be individualized and tailored to the patient's clinical scenario. Conventional treatment consists of transfusions, iron chelation, splenectomy, supportive therapies, and psychological support. Nonconventional treatment includes hematopoietic stem cell transplantation which remains to be the only curative treatment, fetal hemoglobin modulation, and gene therapy [2, 4, 5, 9].

In this review, we shall cover two of the major complications encountered in *β*-TI: endocrine and bone complications. We will also shed light on iron overload in *β*-TI as well as other mechanisms that may lead to these complications.

## 2. Iron Overload in *β*-TI

Iron overload in *β*-TI is multifactorial and attributed primarily to increased gastrointestinal iron absorption [10]. It can also result from chronic hemolysis and occasional blood transfusions required to treat disease complications [10, 11]. In response to chronic anemia and ineffective erythropoiesis, levels of growth and differentiation factor 15 (GDF 15), twisted gastrulation factor 1, and hypoxia-inducible transcription factors (HIFs) increase leading to hepcidin suppression and ferroportin and erythropoietin upregulation [11–14]. The outcome is an increase in duodenal iron absorption and release of iron from the reticuloendothelial system [11]. Increased gastrointestinal iron absorption, coupled with transfusions, leads to iron overload. Even though iron overload occurs in patients with *β*-TI at a much slower rate than that in those with regularly transfused *β*-TM, with advancing age, it can reach levels much higher than normal thresholds with markedly elevated liver iron concentration (LIC) and high levels of circulating toxic non-transferrin-bound iron (NTBI).

Serum ferritin levels and LIC determined by R2 and R2^*^ magnetic resonance imaging (MRI) positively correlate in *β*-TI patients [15–17] where 800 and 300 ng/mL of serum ferritin correspond to 5 mg and 3 mgs Fe/g dry weight (DW) [18]. Vascular, endocrine, and bone morbidity in *β*-TI has been shown to be significantly associated with serum ferritin more than 800 ng/mL and LIC more than 6-7 mg Fe/g DW [18–21]. Spot ferritin measurements, however, may underestimate the burden of iron overload and subsequently delay therapy [20]. LIC, which is the reliable and noninvasive gold standard, approximates iron overload better than serum ferritin [16, 20, 22, 23].

If untreated, iron overload will lead to organ dysfunction involving mostly the liver, heart, and endocrine organs and a wide spectrum of complications and clinical outcomes. A recent study (THALASSA) on 95 patients with NTDT showed efficacy of the once daily oral iron chelator deferasirox in patients at least 10 years of age with LIC ≥ 5 mg Fe/g DW and serum ferritin of at least 800 ng/mL [18]. Despite the availability of chelation therapy including oral agents, iron overload remains a problem because of poor adherence to chelation regimens and high cost of such treatment.

## 3. Endocrine Complications in *β*-TI 

Endocrine complications are amongst the most common complications in *β*-TI and are mostly attributed to iron overload and suboptimal chelation [2–4, 6, 24]. They are associated with splenectomy, increasing age, severe ineffective erythropoiesis, and low fetal hemoglobin levels [9, 24]. The frequency of these complications is lower than that in *β*-TM and varies greatly according to severity of the anemia and iron overload [25]. Earlier onset of these complications is observed with higher LIC compared to lower concentrations [21] and a lower frequency has been observed in patients on iron chelation therapy and or on hydroxyurea [9]. However, no relationship could be established between endocrine dysfunction and serum ferritin level, age of start of desferrioxamine, and hemoglobin level [26, 27].

The most frequent endocrine complications reported in *β*-TI are growth retardation, delayed puberty, hypogonadism, diabetes, impaired thyroid, parathyroid and adrenal functions, and dyslipidemias. Early recognition and treatment of endocrine complications is important in order to prevent late irreversible sequelae and increase the chances of successful reproduction. Patients with established endocrine disease should be referred to an endocrinologist and managed according to recommendations in *β*-TM patients [2, 4, 6, 24, 26, 27].

The sections below will offer a detailed up to date review of the most frequent endocrine complications encountered in *β*-TI. They will also summarize important recommendations for screening and management of these complications and highlight the 2013 Thalassemia International Federation (TIF) Guidelines for the Management of NTDT [24].

### 3.1. Growth Retardation

The prevalence of short stature in children and adults with thalassemia is approximately 25% regardless of the type of the thalassemia and serum ferritin concentration [25]. 20%–30% of thalassemic patients have growth hormone (GH) deficiency and 70–80% have peak growth hormone (GH) levels on provocative tests lower than those seen in patients with constitutional short stature [28]. In *β*-TI, growth hormone deficiency is seen in 31% of patients (28) while the prevalence of short stature (height more than 2 SD below man height for age (below 3rd percentile)) varies between reports ranging from 7 to 46% [26, 27]. In children and adolescents, hypogonadism has been shown to be associated with short stature and GH deficiency to be a significant negative predictor of height. In adults, GH deficiency was the only significant predictor of short stature [25].

The pathogenesis of growth failure in thalassemia is multifactorial and is mainly due to transfusional iron overload and resulting endocrinopathies (GH deficiency, hypothyroidism diabetes), nutritional deficiencies, and intensive use of chelating agents particularly desferrioxamine. Other aetiologies particularly in suboptimally treated children are increased metabolism, chronic anemia, and hypoxia. The anterior pituitary is particularly sensitive to iron associated free radical oxidative stress. Even a modest amount of iron deposition in the anterior pituitary by MRI can interfere with its function. Dysregulation of the GH insulin like growth factor axis leads to growth hormone deficiency and growth deceleration [25, 28].

All patients with NTDT including those with *β*-TI who are ≥10 years should undergo standing and sitting height every 6 months, bone age, growth hormone stimulation, insulin-like growth factor (IGF)-1 level, and IGF-BP3 level (in patients who fall-off the growth curve (5%) and have decreased height velocity or delayed bone age, desferrioxamine toxicity, and other hormonal and nutritional imbalances) [24].

Frequent transfusions should be considered in patients with growth failure (height is more indicative of growth pattern than weight) with reassessment for tapering or withdrawal when a sustained clinical benefit is achieved [24].

### 3.2. Delayed Puberty and Hypogonadism

Delayed puberty and hypogonadism are the most common endocrine common complications in *β*-TI and are attributed to iron-mediated damage leading to dysregulation of the hypothalamic-pituitary axis [9, 25, 26, 28–30]. Delayed puberty is defined as no puberty in girls by 13 years and in boys by 14 years. Hypogonadism is defined as absence of testicular development in boys and breast development in girls by 16 years. Hypogonadic hypogonadism is the most frequent and often undertreated endocrine complication of *β*-TI, seen in 24% of patients, affecting females more than males [9, 25, 30]. It has been shown to be correlated with early onset of transfusion therapy and serum ferritin levels of approximately 2000 ng/mL in TM patients [31] and high LIC [21], increasing age [25] and HU treatment [9] in *β*-TI patients. It can lead to maternal and fetal problems even when iron chelation therapy is employed [32–35]. However, gonadal function of women with this complication is not affected and fertility can be salvaged [36].

Routine early screening for delayed puberty and hypogonadism is warranted to initiate treatment and prevent complications. Tanner staging every 6 months for prepubescent children and annual evaluation of luteinizing hormone, follicular stimulating hormone, insulin-like growth factor (IGF), and IGF-binding protein-3 for children between 8 and 10 years are recommended [37]. Testosterone, estradiol, pelvic ultrasound, nutrient levels, and thyroid function are also indicated for patients with evidence of pubertal delay. Routine assessment for infertility, secondary hypogonadism, and impotence needs to be performed in all adults [24].

Literature still lacks accurate management guidelines for delayed puberty and hypogonadism in *β*-TI. Gonadal steroids (ethinyl estradiol, estrogen-progesterone hormone, and testosterone esters) and gonadotropin releasing hormone should be initiated for females >13 and males >16 years of age not showing pubertal change [7, 25, 37]. Human chorionic gonadotropin (hCG), human menopausal gonadotropin (hMG), and intracytoplasmic sperm injection (ICSI) can be offered to males with spermatogenesis problems to assist in attaining pregnancy in the partner [7]. In females with chronic anovulation, stimulation with gonadotropins can still increase estradiol and produce ova, but global assessment of the patient is necessary before induction of pregnancy [38].

### 3.3. Fertility and Pregnancy

In most *β*-TI patients, fertility is not affected and most pregnancies can be achieved spontaneously [38, 39]. Pregnancy in this patient population is, however, associated with intrauterine growth retardation (IUGR) in more than half of cases, higher risk of abortion, preterm and cesarean section delivery, and thromboembolic events [40, 41]. This is attributed to multiple factors including anemia, hypoxia, acute hypersplenism, splenomegaly, and hypercoagulability state [40]. Splenectomy may be required after or even during gestation. With advancing pregnancy, anemia increases and blood transfusion therapy may be needed but it is often limited by the risk of alloimmunization in previously untransfused women [42]. Increased risk of thromboembolism may necessitate short-term anticoagulation with low-molecular weight heparin and platelet anticoagulants followed by a long-term oral anticoagulant [5, 40]. Folic acid deficiency due to increased erythropoiesis, poor absorption, and low dietary intake is common and folic acid supplements are recommended to prevent fetal neural tube defects [5, 11, 43]. Optimal management of pregnant *β*-TI women, therefore, requires a multidisciplinary approach with close maternal and fetal surveillance. Assessment of iron overload, cardiac, endocrine, liver, viral, and red blood cell antibodies status before pregnancy and ensuring adequate management are recommended [24].

### 3.4. Diabetes Mellitus and Glucose Intolerance

Glucose tolerance abnormalities and diabetes mellitus are common complications in thalassemia patients. While glucose intolerance occurs at an earlier stage during adolescence in *β*-TI patients, diabetes frequently occurs at later stages and is usually secondary to iron overload and subsequent chronic liver disease [34, 44]. The prevalence of diabetes and glucose intolerance is 9.4% [34] and 7.1% [34, 44] in *β*-TM and 2% and 24% in *β*-TI [26, 27].

The development of diabetes in thalassemia is attributed to impaired insulin excretory function secondary to chronic iron overload in the pancreas [45], selective immune system activation against pancreatic *β*-cells leading to cell damage [46], and/or pancreatic cell death due to fat transformation [47]. In addition, Noetzli et al. (2012) showed that impaired insulin sensitivity was associated with inflammation markers and somatic iron overload [48]. Even though several studies correlated elevated LIC with development of diabetes [49, 50], a single measurement of LIC is a poor predictor of endocrine failure, especially pancreatic iron deposition [51, 52]. A pancreatic MRI T2^*^ coupled with a gradient echo sequence is recommended for detecting pancreatic fat and predicting the incidence of diabetes [52]. A serum ferritin level of around 3000 ng/mL has been shown to be associated with a higher risk of developing diabetes [31].

Iron-mediated diabetes can be partially reversed if treated earlier [53]. High doses of insulin are required to correct blood glucose levels in these diabetic patients [50]. Experts recommend early screening and detection of glucose impairments and insulin resistance in all thalassemia patients starting at age of 8 to 10 years as the disease can be halted before developing into overt-diabetes in adulthood [25, 43]. According to TIF NTDT guidelines, patients with *β*-TI who are ≥10 years should undergo annual fasting blood sugar and if indicated oral glucose tolerance test [24].

### 3.5. Dyslipidemia

Lipid profile is altered in patients with *β*-TM and *β*-TI [54]. Serum total cholesterol (TC) and low-density lipoprotein cholesterol (LDL-C) levels are lower in thalassemia patients than normal controls, while triglycerides (TG) levels are not significantly different or elevated compared to normal controls [54–58]. In *β*-TI patients, lipid profile variations are common but not a troubling feature of the disease. A study conducted by Hartman et al. has revealed that the levels of TC, HDL-C, and LDL-C were significantly lower among children and adolescents with *β*-TI compared to *β*-TM and healthy controls [54]. These values were independent of age, sex, ferritin, and hemoglobin levels [54]. The mechanism and pathophysiology behind such findings are still unclear and it is hypothesized that in *β*-TI, lipid profile changes are secondary to continuous erythropoietic activity and elevated cholesterol consumption [51, 59]. In *β*-TM, on the other hand, oxidative stress and high iron overload appear to be the main contributors [56, 60]. Liver damage may also play an important role in determining the altered lipoprotein pattern in beta-thalassemia [57]. In addition, vitamin E plays an important role in the reduction of LDL oxidation as they were both correlated. A study conducted on 30 individuals with TI has shown that the content of plasma and LDL *α*-tocopherol was significantly lower as compared to the control group [61]. While the studies showing the efficacy of vitamin E are limited to 15 patients with TI that were treated with 600 mg/day for a period of 9 months, the levels of vitamin E started to increase within 3 months as well as the levels of LDL [62].

Routine lipid profile investigation in young *β*-TI patients is not recommended [54]. More investigations are, however, required to show the basic mechanism of the dyslipidemia in this thalassemia subgroup and whether calibrating serum lipid levels may affect the disease outcome.

### 3.6. Hypothyroidism and Hypoparathyroidism

Hypothyroidism is a late consequence of iron deposition in the thyroid gland that ultimately leads to parenchymal fibrosis [63]. Prevalence of hypothyroidism ranges from 4% to 24.4% [27, 31, 43, 63, 64] in *β*-TM and 2 to 3% in *β*-TI [11]. Splenectomy is a specific risk factor for hypothyroidism in *β*-TI. In TM, contributing factors include poor compliance with desferrioxamine and elevated serum ferritin levels reaching 3000 ng/mL, which is correlated with hypoparathyroidism as well [31]. The risk of hypothyroidism is significantly increased with every 1 mg Fe/kg DW elevation in LIC. According to the TIF NTDT guidelines, free thyroxine (FT4) and thyroid-stimulating hormone need to be performed annually on all *β*-TI patients ≥10 years [24]. Hypothyroidism must be treated promptly with L-thyroxine [7].

Hypoparathyroidism, seen in up to 6.7% of TM patients, is not well studied in *β*-TI [65]. *β*-TI patients ≥10 years need to be screened for this complication by calcium, phosphate, and vitamin D every year and by parathyroid hormone level if indicated [24]. Calcitriol is recommended for mild hypocalcemia and intravenous calcium administration followed by oral vitamin D for severe cases with tetany [7].

Early iron chelation to prevent hypothyroidism and hypoparathyroidism is recommended by the Thalassemia Clinical Research Network. Thyroid dysfunction, if detected early, can also be reversed with combined desferrioxamine (DFO) and deferiprone (DFP) chelation [65]. In a study from North America, ninety-one percent of chelated patients showed no thyroid dysfunction and 96% were devoid of hypoparathyroidism [32]. In the Optimal Care Study, hydroxyurea (HU) treatment was found to be protective against hypothyroidism in *β*-TI when compared to transfusion therapy, which was found to be a risk factor [9, 66]. However, low doses of HU (8–15 mg/kg/day) did not alter thyroid function in *β*-TI patients in one study [66]. More studies are required to elucidate the role of HU in the thyroid function of *β*-TI patients.

### 3.7. Hypoadrenalism

Contradictory data exist regarding the pituitary-adrenal dysfunction in thalassemia patients. Some studies described normal basal concentrations of aldosterone, cortisol and adrenocorticotropic hormone (ACTH) and normal response to stimulation by ACTH and metyrapone [67–69]. Other studies reported adrenal hypofunction, elevated basal ACTH levels, and increased response to insulin-induced hypoglycemia [70–73]. In their study, Huang et al. (2014) found that 61% of TM patients had adrenal insufficiency, predominantly among males (92%). Ten out of eleven subjects possibly had insufficiency attributed to a hypothalamic origin [74]. Impaired adrenal function is attributed to iron loading of the pituitary and the adrenal and may play an important role in determining the delayed sexual maturation almost always present in the thalassemic patients [72, 73, 75]. All *β*-TI patients ≥10 years should undergo annual adrenocorticotropic hormone stimulation test [24]. Studies on the pituitary-adrenal axis function specific to *β*-TI as well as treatment guidelines for those with adrenal insufficiency are still lacking.

### 3.8. Bone Abnormalities

Bone abnormalities in *β*-TI are quite frequent and range from a decrease in the bone mineral density (BMD) and consequent osteoporosis to spinal cord compression and increased risk of the development of fractures. They are similar to those seen in *β*-TM but often more marked due to enhanced ineffective erythropoiesis. The underlying cause of the bone disease in *β*-TI is attributed to several factors including bone marrow expansion, ineffective erythropoiesis, vitamin D deficiency, genetic factors, endocrine dysfunctions secondary to iron overload, and reduced physical activity [9, 76, 77].

The majority of patients with *β*-TI have decreased levels of IGF-1 that usually plays an essential role in the bone remodeling cycle that stimulates osteoclasts and the differentiation and proliferation of osteoblasts. An increased level of the receptor activator of nuclear factor KB ligand (RANKL) leads to decreased bone thickness followed by bony deformities, osteopenia, and ultimately fractures [78]. Additionally, the* Bsml* Vitamin D receptor (VDR) gene has been shown to be linked with the osteopenia that develops in thalassemia [79]. The urinary excretion of urinary N-telopeptide cross-linked collagen type I (NTx) has been shown to be a sensitive and reliable index of the hip BMD Z-score in patients with thalassemia [79].

### 3.9. Osteoporosis

Osteoporosis is defined by WHO as a decrease in the bone mineral density and disruption of the bone architecture leading to an increased risk of fractures [80]. A decrease in bone mass can occur due to increased bone resorption or decreased bone formation both of which can lead to osteopenia/osteoporosis in thalassemia [81]. Factors that have been associated with increased rates of osteoporosis in *β*-TI patients include female gender, iron overload, splenectomy, and low fetal hemoglobin levels [9, 19–21]. A recent study has shown that there is a significantly higher rate of osteoporosis in *β*-TI as compared to *β*-TM, accounting for 81.6% and 59.8%, respectively [82]. The prevalence of osteopenia was lower in *β*-TI as compared to *β*-TM accounting for 8% and 22.6%, respectively [82]. Furthermore, a reduction of BMD was present in the spine, femoral neck, and distal radius in more than 2/3 of the patients with both *β*-TM and *β*-TI [81]. The decrease that was detected in the lumber region was significantly linked with the level of hemoglobin suggesting that the lumbar region is mostly affected among thalassemia patients [83].

Several procedures have been used to describe the extent of bone loss in patients with thalassemia including the Dual energy X-Ray absorptiometry (DXA) to estimate the BMD and the peripheral Quantitative Computer Tomography (pQCT) to assess the regional changes of BMD [84–86]. In a study comparing the use of pQCT with DXA in patients with thalassemia, a reduced pQCT of the trabecular and cortical parameters was found in *β*-TI and was more severely affected than in *β*-TM [85]. The recent TIF NTDT guidelines recommend that *β*-TI patients ≥10 years be screened for osteoporosis by annual BMD of the spine, hips, radius, and ulna (dual-energy X-ray absorptiometry) and undergo hormonal and nutritional profile and spine imaging for back pain or neurological findings [24].

Studies on the prevention and management of osteoporosis in *β*-TI are scarce. Lower rates of osteoporosis have been reported in *β*-TI patients treated with iron chelation and hydroxyurea than in those who have not [9]. Adherence to daily exercise programs can help maintain bone strength and improvement of the bone status aiding in prevention of the bone complications. Despite transfusion normalizing hemoglobin levels, iron chelation, and adequate hormonal replacement therapy, patients with thalassemia can continue to have progressive bone disease and BMD loss over time [82, 85, 87].

Vitamin D and calcium are often prescribed to patients with *β*-TI with careful renal function monitoring in the hope of improving mineral density. The efficacy and exact treatment regimen for these supplements have not yet been defined [5, 42]. Bisphosphonates, which are potent osteoclast inhibitors, constitute the treatment of choice in thalassemia associated osteoporosis as these drugs modify the biochemical markers of bone formation and resorption. They have been shown to be safe and efficacious in improving BMD and reducing bone complications and pain in both *β*-TM and *β*-TI [5, 42, 88–92]. Dental surveillance is necessary during treatment with bisphosphonates, because this treatment has been associated with jaw necrosis. The 3 most well studied bisphosphonates in thalassemia are zoledronic, pamidronate, and neridronate [88–92]. Further studies are still, however, required to establish the long-term efficacy and outcome of bisphosphonates. The continuous increase in erythropoietin activity, despite treatment with zoledronic acid and the increase in BMD, contributes to the bone loss in *β*-TI suggesting that blood transfusions may be capable of controlling bone loss more efficiently than bisphosphonates [93]. There is a definite need for more research studies to determine the value of medications such as parathyroid hormone treatment, denosumab, and sotatercept for the treatment of osteoporosis in *β*-TI patients [94].

### 3.10. Fractures

Fractures are more frequently seen in *β*-TM compared to *β*-TI and the site of fractures differs with arms and forearms affected in *β*-TM and metacarpal bones in *β*-TI [95]. The prevalence rate of fractures in *β*-TI is 12.2% and is likely to increase with age and in patients with a lower lumbar bone mass at a rate almost similar to that seen in *β*-TM. A decreased BMD is a major risk factor for the development of fractures in *β*-TI. Other independent risk factors are the use of sex hormone replacement therapy and hypogonadism [96].

### 3.11. Extramedullary Hematopoiesis

Extramedullary hematopoiesis (EMH), defined as the development of erythropoietic tissue outside the marrow cavity, is a phenomenon that compensates for the decreased efficiency of the bone marrow in providing RBCs for the circulation [2]. Many body sites can be involved in EMH including the spleen, lymph nodes, liver, breast, spinal canal, prostate, heart, thymus, kidney, and adrenal glands [2, 4, 9]. The incidence of EMH is highest among individuals with a chronic hemolytic anemia, specifically NTDT patients [2], thus contributing to the development of osteoporosis and deformities of the facial bones, obliteration of maxillary sinuses, and protrusion of the upper jaw, along with increased risk for fractures of long bones and spinal compression [6, 97, 98]. The incidence of EMH in patients with *β*-TI may reach up to 20% compared to poly-transfused TM patients where the incidence remains less than 1% [2]. However, more than 80% of cases may remain asymptomatic and the lesions are usually discovered incidentally by radiologic techniques [2, 4, 6, 97].

One of the devastating complications of EMH is its progression to a spinal cord compression, secondary to a paraspinal mass that can manifest as paraplegia or cauda equina syndrome [99–101]. Among all imaging modalities, MRI is the method of choice for the diagnosis and follow-up evaluation of a spinal cord compression due to an EMH [99, 101–103]. Establishing an early diagnosis of *β*-TI and instituting optimal management of the underlying anemia and associated symptoms are important steps to prevent EMH and its complications.

The treatment for spinal cord compression involves blood transfusions, surgical decompression, and radiotherapy either singly or in combination [2, 4, 6]. Though hypertransfusion is effective in relieving compression symptoms, HU may replace transfusions and limit expansion of the ectopic hematopoietic tissue [104]. While surgical decompression is an effective method that allows improvement of symptoms, radiotherapy alone remains to be the treatment of choice. The latter cannot be performed until a diagnosis is made. Radiotherapy hinders the hematopoietic activity thereby triggering the decline in the size of the mass and improvement of the associated symptoms [100].

## 4. Conclusion and Recommendations

Recent research has revealed that *β*-TI is not a mild disease and is associated with greater morbidity and a wider spectrum of organ dysfunction and complications than previously thought. Endocrine and bone complications are highly prevalent in this disease and necessitate close monitoring, treatment, and follow-up. Early recognition of these complications, institution of appropriate treatment including transfusion regimen and chelation therapy, and specific treatment of each complication are the keys to successful management. Meticulous follow-up by a multidisciplinary team in a comprehensive thalassemia center as well as strict compliance with tailored treatment protocols are major prerequisites for achieving and maintaining an excellent prognosis. The main challenges that remain are the under recognition and underestimation of the morbidity of such complications as well as the absence of affordable worldwide-established treatment protocols.
